# Cerebral venous thrombosis caused by spontaneous intracranial hypotension due to spontaneous spinal cerebrospinal fluid leakage in the high cervical region: a case report

**DOI:** 10.3389/fneur.2023.1256200

**Published:** 2023-10-26

**Authors:** Man Li, Yi Li, Liwen Tai, Hui Li, Li Qing Wang, Yue Li Zou, Wen Feng Feng, Yue Liu, Xiaopeng Liu, Jun Ying He

**Affiliations:** ^1^Department of Neurology, Second Hospital of Hebei Medical University, Shijiazhuang, China; ^2^Second Hospital of Hebei Medical University, Shijiazhuang, Hebei Province, China; ^3^Department of Neurosurgery, Second Hospital of Hebei Medical University, Shijiazhuang, Hebei Province, China

**Keywords:** cerebral venous thrombosis, epidural blood patch, low cranial pressure, spontaneous cerebrospinal fluid leak, spontaneous intracranial hypotension

## Abstract

Spontaneous intracranial hypotension (SIH) may lead to cerebral venous thrombosis (CVT). This case report describes the diagnostic and treatment processes used for a patient with CVT caused by SIH due to spontaneous spinal cerebrospinal fluid (CSF) leakage in the high cervical region. Clinical data were collected from a 37-year-old man with an initial symptom of spontaneous posterior cervical pain. The diagnostic and treatment processes of SIH-induced CVT were described. A magnetic resonance imaging (MRI) study showed superior sagittal sinus thrombosis, and a lumbar puncture revealed a low initial CSF pressure of less than 60 mmH_2_O. The patient underwent anticoagulation and fluid rehydration therapies. No abnormalities were observed in the thoracic MRI scan, but a cervical MRI scan revealed a spontaneous CSF leak. An epidural blood patch with autologous blood was performed, and symptoms completely resolved 3 days after the procedure. This report proposes a diagnostic procedure for detecting rare cases of SIH-induced CVT, thereby preventing future misdiagnoses and delayed treatment. When a patient presenting with CVT in conjunction with intracranial hypotension has no history of trauma or piercing, SIH caused by spontaneous spinal CSF leakage should be considered as a potential cause of secondary low intracranial pressure. For detection of CSF leaks at rare sites, an MRI of the whole spine rather than a localized MRI of the spine needs to be performed to avoid misdiagnosis. An epidural blood patch should be performed as soon as possible as it may shorten the length of hospitalization and improve prognosis.

## Introduction

Cerebral venous thrombosis (CVT) is a rare neurological disease that may cause life-threatening complications, such as epilepsy, cerebral hemorrhage, and cerebral herniation. The incidence of CVT is approximately five cases per 1,000,000 people per year (1). The major risk factors for CVT are oral contraceptives, hereditary thrombosis, pregnancy, puerperium, and systemic diseases (e.g., tumors, autoimmune diseases, and infections).

CVT induced by spontaneous intracranial hypotension (SIH), a rare cause of CVT occurring in only 2% of SIH cases, is characterized by postural headache and a cerebrospinal fluid (CSF) pressure of less than 60 mmH_2_O (2). Although the mechanisms of SIH-induced CVT are not fully understood, three hypotheses have been proposed (2): (1) Following the logic of the Monro–Kellie (3) doctrine, the loss of cerebrospinal fluid leads to compensatory dilation of veins, which slows down blood flow through the straight sinus, a complication that has been reported in some cases of CVT. (2) Through an epidural incision, CSF flows into the epidural space rather than into the venous system, resulting in increased blood viscosity in the epidural veins (2). (3) The sagging of brain tissues can pull on the parenchymal veins, leading to turbulence or stagnation of the venous blood flow. The most common focal brain injuries of SIH-induced CVT are cerebral venous infarction, cerebral hemorrhage, subarachnoid hemorrhage and focal cerebral edema, and the most common symptoms are epilepsy and limb weakness (4–6).

Spontaneous spinal CSF leakage occurs when there is a hole or tear in the membrane surrounding the dura. Dura holes or tears can cause a single localized CSF leak or multiple simultaneous leaks, either of which may lead to SIH. The annual incidence of spontaneous spinal CSF leakage is approximately four cases per 100,000 people (7). Spontaneous spinal CSF leakage is more common in middle-aged women (8). The most common clinical manifestation of spontaneous spinal CSF leakage is orthostatic headache; however, the pathogenic mechanisms remain unclear. A lack of understanding about the role of spontaneous spinal CSF leaks in SIH may lead to delayed diagnosis and treatment, thus increasing the risk of other complications, such as subdural effusion, subdural hematoma, and even life-threatening CVT.

In this case report, we present a complex case with CVT secondary to SIH caused by a spontaneous spinal CSF leak in the high cervical region.

A 37-year-old man experiencing frontal and posterior neck pain while sitting but without any inducement was admitted to the Second Hospital of Hebei Medical University 5 days after symptom onset. The pain severity became aggravated when the patient was in the sitting or standing position but was alleviated in the decubitus position. He was diagnosed with cervical spondylosis in the local hospital, but his headache became progressively worse. One day before the occurrence of left upper and lower limbs weakness and numbness, the patient had a sudden seizure and lost consciousness. There were three seizures in total, each lasting about 1 min. After emergency treatment, including sedation and seizure control, the convulsion was relieved and consciousness was recovered. Subsequently, the patient followed a continuous regimen of valproate sustained-release tablets orally (500 mg, twice daily), and no convulsion was observed.

The patient had a history of upper respiratory tract infection in the seven days prior to admission, but no history of chronic disease or surgical trauma. He also had no family history of genetic diseases. The patient presented with left hemiplegia. Physical examination revealed a neck stiffness and active tendon reflex in the left. The left Babinski sign is positive.

**Figure 1:**
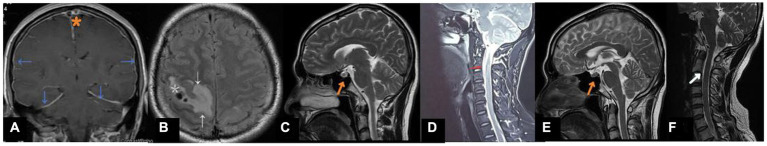
**(A)** Coronal T1 with contrast shows thrombus in the superior sagittal sinus and in a cortical vein at the vertex (*). Note diffuse thickening and enhancement of the dura (blue arrows). **(B)** FLAIR image showing right parietal lobe hemorrhage (*)and edema (arrow)resulting from the venous infarct. **(C)** At first resentation. Sagittal T2-weighted MRI showed full pituitary gland enlargement(arrows). **(D)** Sagittal T2-weighted MRI revealed ventral C1-C2 CSF accumulation(arrows). **(E)** 3-Month follow-up.The pituitary gland has decreased in size(arrows). **(F)** 3-Month follow-up.Sagittal T2-weighted MRI of the spine showed a significant decrease in cerebrospinal fluid collection.

A head magnetic resonance imaging (MRI) + diffusion weighted imaging (DWI) + magnetic resonance angiography (MRA) study showed enlargement of the pituitary gland, descent of the cerebellar tonsils and brainstem, and crowding of the posterior fossa (Figure 1C). An electroencephalogram showed increased slow wave activity. An enhanced brain MRI scan showed hemorrhagic foci in the right parietal lobe with surrounding edema (Figure 1B), filling defects of the superior sagittal sinus, cystic signal shadow under the right top cranial plate, and diffuse enhancement of the dura and pia mater (Figure 1A). Multiple filling defects at the top and back of the superior sagittal sinus (Figure 1A) were shown on a brain magnetic resonance venogram (MRV), suggesting suggesting venous sinus thrombosis venous sinus thrombosis. After admission, a lumbar puncture was performed, revealing an initial CSF pressure of less than 60 mmH_2_O, indicating intracranial hypotension. The white blood cell count was 11.70 × 10^9^/L, and the absolute value of the neutrophil was 8.50 × 10^9^/L. The D-dimer was 0.70 μg/mL (↑). The results of a routine CSF examination were normal. Biochemical examination of the CSF showed that the protein content was 2.46 g/L (↑), and the glucose and chlorine levels were normal. The patient was treated with nadroparin calcium (anticoagulant therapy), acyclovir and foscarnet sodium (antiviral therapy), and cefoperazone sodium/sulbactam sodium (anti-infection therapy) *via* injection, as well as intravenous rehydration therapy. Symptoms of headache and left limb weakness were slightly alleviated by the treatment but did not completely resolve, and the patient was still unable to walk. Further MRI study of the cervical and thoracic vertebrae showed CSF collection at the C1-C2 level (Figure 1D), suggesting a dural CSF leak. Therefore, a percutaneous epidural blood patch was performed under the guidance of computerized tomography (CT). The headache was completely relieved 3 days after the procedure, and the patient was able to walk freely. One week after the procedure, a cervical MRI showed that the fluid collection was significantly smaller. Re-examination with an MRV showed no filling defect. Three months later, the pituitary gland returned to normal (Figure 1E). The spinal CSF collection was significantly reduced (Figure 1F). The patient was followed up by telephone for 4 months. At the end of the follow-up period, he reported no headache or convulsion and that his body movement had returned to normal.

## Discussion and conclusions

The pathogenic mechanisms of spontaneous CSF leaks are still unclear. In some patients with a spontaneous spinal CSF leak, there may be an underlying connective tissue disorder. It is generally believed that there is a structural vulnerability point on the spinal membrane that is more susceptible to holes or tears (9). Mechanical injuries, such as trauma, may cause CSF leaks from the dura mater at the spinal level (10, 11). Generalized connective tissue disease is also considered a cause of spontaneous CSF leaks (12, 13). The common clinical manifestation of SIH induced by spontaneous spinal CSF leaks is postural headache. However, whether this headache is caused by reduced CSF volume or low intracranial pressure remains unknown. Since headache is a common symptom in patients with neurological disorders, spontaneous spinal CFS leaks are often misdiagnosed (14). Recently, Jones et al. proposed a diagnosis and treatment procedure for SIH induced by spontaneous spinal CSF leaks (15): (1) Perform cranial MRI scan + contrast. (2) If the brain MRI shows dural enhancement and downward shift of brain structures, a non-targeted epidural blood patch should be performed, followed by general treatments (i.e., bed rest and symptomatic treatment with acetaminophen, butalbital, and caffeine). (3) If the symptoms persist for 2 to 3 weeks, a plain MRI scan (without enhancement) of the spine and CT myelography should be performed. (4) If there is a localized leak, a targeted epidural fibrin patch should be performed. (5) If the symptoms persist for 2 to 3 weeks, surgical suturing should be considered for lumbar CSF leakage.

The imaging results of our patient after admission showed superior sagittal sinus thrombosis and an initial CSF pressure of lower than 60 mmH_2_O. Additionally, he had a positional headache but no history of trauma or piercing, indicating SIH. Further cerebral MRI scan confirmed the diagnosis. Most spontaneous CSF leaks occur at the thoracic level (16). However, no abnormality was observed in the thoracic MRI scan of this patient. Further cervical MRI detected ventral CSF collection in the C1-C2 segment of the neck, indicating a localized CSF leak (17, 18). Performing an epidural blood patch has been shown to effectively relieve symptoms (e.g., headache) (19). Cho et al. reported that a site-directed epidural blood patch effectively alleviated symptoms in 87.1% of 56 patients, whereas a blind epidural blood patch through the epidural approach to the lumbar spine or upper chest was effective in 52% of the patients (20). In our case, a CT-guided epidural blood patch was performed, but the patient’s symptoms did not completely resolve after administering an initial treatment to improve circulation, antiviral treatment, anticoagulant treatment, anti-infection therapy, and fluid replacement. Three days after the epidural blood patch procedure, symptoms completely resolved. At the end of a 4-month follow-up period, the patient reported no headache or convulsion and that his body movement had returned to normal.

When a patient has CVT in conjunction with intracranial hypotension but has no history of trauma or piercing, SIH should be considered. In cases of spontaneous spinal CSF leaks at rare sites, an MRI of the whole spine rather than a localized MRI of the spine should be performed to avoid misdiagnosis. In these patients, an epidural blood patch should be performed as soon as possible, which may shorten the length of hospitalization and improve prognosis.

## Data availability statement

The original contributions presented in the study are included in the article/supplementary material, further inquiries can be directed to the corresponding authors.

## Ethics statement

The studies involving human participants were reviewed and approved by the Ethics Committee of the Second Hospital of Hebei Medical University. Written informed consent from the patients/participants or patients/participants' legal guardian/next of kin was not required to participate in this study in accordance with the national legislation and the institutional requirements. Written informed consent was obtained from the individual(s) for the publication of any potentially identifiable images or data included in this article.

## Author contributions

ML: Writing – original draft. YLi: Writing – review & editing. LT: Writing – review & editing. HL: Investigation, Writing – review & editing. LW: Investigation, Writing – review & editing. YZ: Validation, Writing – review & editing. WF: Data curation, Writing – review & editing. YLiu: Investigation, Writing – review & editing. XL: Resources, Writing – review & editing. JH: Writing – review & editing.
